# Treatment patterns associated with Duloxetine and Venlafaxine use for Major Depressive Disorder

**DOI:** 10.1186/1471-244X-11-19

**Published:** 2011-01-31

**Authors:** Wenyu Ye, Yang Zhao, Rebecca L Robinson, Ralph W Swindle

**Affiliations:** 1 Lilly USA, LLC, Indianapolis, Indiana, USA; 2 Global Health Outcomes, Eli Lilly and Company, Indianapolis, Indiana, USA

## Abstract

**Background:**

Duloxetine and venlafaxine extended release (venlafaxine XR) are SNRIs indicated for the treatment of MDD. This study addresses whether duloxetine and venlafaxine XR are interchangeable in their patterns of use with patients who are depressed or are used more selectively based on treatment history, background characteristics, and presenting symptoms.

**Methods:**

This was a retrospective analysis of an administrative insurance claims database. We studied patients in managed care with major depressive disorder (MDD) treated with duloxetine or venlafaxine XR. Predictors of treatment and cost were assessed using Chi-square and logistic regression analyses of demographics and past-year medication use and comorbidities.

**Results:**

Patients with MDD treated with duloxetine (n = 9,641) versus venlafaxine XR (n = 8,514) tended to be older, slightly more likely to be female, and treated by a psychiatrist (*P *< 0.0001). In the prior year, more duloxetine patients (vs. venlafaxine XR) received ≥3 unique antidepressants (20.8% vs. 16.6%), ≥3 unique pain medications (25.5% vs. 15.6%), and had ≥8 unique diagnosed comorbid medical and psychiatric conditions (38.6% vs. 29.1%). The prior 6-month total health care costs were $1,731 higher for duloxetine than for venlafaxine XR and declined for both medications in the 6 months after treatment began. Logistic regression analysis revealed that 61% of duloxetine patients and 61% of venlafaxine XR patients were predictable from prior patient and treatment factors.

**Conclusions:**

Patients with MDD treated with duloxetine tended to have a more complex and costly antecedent clinical presentation compared with venlafaxine XR patients, suggesting that physicians do not use the medications interchangeably.

## Background

Selective serotonin-reuptake inhibitors (SSRIs) and serotonin norepinephrine-reuptake inhibitors (SNRIs) are mainstays in the pharmacologic management of major depressive disorder (MDD) in the United States [1]. SSRIs, such as sertraline, paroxetine, fluoxetine, and escitalopram/citalopram, have been used for years. However, recent studies demonstrated that fewer than 30% of patients with MDD experience remission with initial SSRI treatment, and approximately 33% of nonremitting patients fail to accept an alternative second treatment [2]. Some clinical studies and meta-analyses suggest that SNRIs may be more effective than SSRIs in ameliorating depressive symptoms in some circumstances [3-5], in achieving greater remission rates [6,7], and in second-line use after poor initial treatment response [8,9]. Data based on analyses of clinical trials are inconsistent, however [10-14]. This study examines the differential real-world use and cost impact of the SNRIs duloxetine hydrochloride and venlafaxine hydrochloride extended release (venlafaxine XR) in the treatment of MDD.

Both duloxetine and venlafaxine XR are SNRIs indicated for the treatment of MDD. Duloxetine and venlafaxine XR have similar mechanisms of action, but duloxetine has a more balanced affinity for both serotonin and norepinephrine transporters, whereas venlafaxine has a higher affinity for serotonin than norepinephrine transporters [15,16]. Clinically, duloxetine has additional pain-related indications for peripheral diabetic neuropathic pain and fibromyalgia [17]. These different pharmacologic and indication profiles may lead practicing physicians to target different types of patients with MDD for different SNRIs.

Many factors may be associated with psychiatrists' selection of an antidepressant. In previous studies, considerations involved in the psychiatrist's selection of an antidepressant included the presence of specific symptoms (52.3%), the presence of a comorbid psychiatric disorder (45.6%), previous treatment response (either positive [17.0%] or negative [25.9%]) [18], previous antidepressant use [19], and sex- and age-related differences [20]. However, little is known about the demographic characteristics, comorbidities, prior medication uses, and health care cost implications of patients initiated on treatment with duloxetine compared with venlafaxine XR. No known studies have compared factors that might predict treatment initiation with one SNRI or the other and the potential impact of differential selection.

Consequently, we sought to examine associations of demographics, prior comorbidities, medication use, and treatment cost, with treatment initiation for the two SNRIs among patients with MDD, using a large US administrative claims database. This study addresses whether these two medications are essentially interchangeable in their actual patterns of use for patients who are depressed or are used more selectively for patients with different kinds of treatment histories, background characteristics, and presenting symptoms.

## Methods

### Data Source and Patient Selection

A retrospective study was conducted using data extracted from a large nationwide US administrative claims database (PharMetrics Integrated Outcomes Database) dating from July 2004 through July 2006. PharMetrics data represent more than 70 different managed-care organizations across the United States and more than 58 million commercially insured patients. The PharMetrics database is Health Insurance Portability and Accountability Act (HIPAA) compliant, de-identified, commercially available to the public, and widely considered exempt from institutional review board (IRB)/ethics committee approval. Due to full data de-identification on the collected data, IRB approvals were neither needed nor sought. The data encompasses comprehensive records on member demographic characteristics, health plan enrollment, inpatient and outpatient services, and prescriptions.

Diagnostic and prescription data were extracted for 12 months before the date of treatment initiation with duloxetine or venlafaxine XR (index date), between July 1, 2005, and July 30, 2006. The index date was defined as the date of the most recent prescription for duloxetine or venlafaxine XR where no prescription for or use of the same medication was present in the prior 3 months. Patients were included in the study if they were commercially insured, 18 to 64 years of age on the index date, and had 1 or more diagnosis of MDD (*International Classification of Diseases*, 9th Edition [ICD-9-CM] codes 296.2x for MDD single episode, or 296.3x for MDD recurrent episode) during the 12 months before the index date. Study patients were also required to have continuous enrollment for the 12 months before the index date. Patients were categorized into two mutually exclusive study cohorts based on the most recent index pharmacy claim: either for duloxetine or venlafaxine XR.

### Study Variables

For patients in each cohort, we used National Drug Codes (NDC) to identify and categorize prior medications in the 12 months before index SNRI initiation, including antidepressants (SSRIs, monoamine oxidase inhibitors [MAOIs]; tricyclic antidepressants [TCAs]); anxiolytics; other psychotropic medications (e.g., antipsychotics, stimulants, atomoxetine, antimanics); sedatives/hypnotics; anticonvulsants; pain-related medications (analgesics, skeletal-muscle relaxants, antimigraine medications); other medications for gastrointestinal, cardiovascular, and respiratory diseases; diabetes mellitus; or allergies. Two additional variables were created to predict initiation of treatment with duloxetine versus venlafaxine XR based on prior uses of antidepressants and pain medications: prior uses for ≥3 unique antidepressants and ≥3 unique pain medications.

Comorbid diagnostic histories were identified based on ICD-9-CM codes in the 12 months leading up to and including the index visit. Medical conditions considered were other depressive disorders (300.4x for dysthymic disorder, 309.1x for adjustment reaction with prolonged depressive reaction, and 311.x for depressive disorder not elsewhere classified), pain, diabetic neuropathy, fibromyalgia, anxiety (classified as generalized anxiety disorder, panic anxiety, post-traumatic stress disorder, social anxiety, and other anxiety disorder), schizophrenia, bipolar disorder, organic psychosis, alcohol dependence, dyslipidemia, hypertension, sleep disorders, gastrointestinal disorders, diabetes mellitus, asthma, heart disease, attention-deficit/hyperactivity disorder (ADHD), drug dependence, and nondependent drug abuse. Specific pain diagnostic subcategories were also identified, including skeletal muscle, back, head, chest, neuropathic pain, irritable bowel syndrome, and other pain conditions not classified elsewhere. (ICD-9 coding groups available from corresponding author on request.) On the basis of prior diagnoses, a predictive indicator variable for patients with ≥8 unique medical disease classes was derived (see Appendix).

To compare health care utilization between the two SNRI cohorts, we calculated total health care costs based on amounts paid by health plans for medical services and prescription medications for 6 months before (and including the index date) and the 6 months after the index date. Medical costs were classified by place of service into inpatient, emergency room, outpatient, and pharmacy costs.

### Statistical Analyses

The following factors were compared for patients in the duloxetine cohort with those in the venlafaxine XR cohort: sociodemographic characteristics (age [mean age and by-group ages 18-35, 36-50, and 51-64 years], gender, plan type, geographic region, and prescriber specialty (psychiatrist or other at the index date), prior medication use, prior medical conditions, and health care claims costs for the patients. Chi-square and Mantel-Haenszel tests were performed for comparisons of categorical variables between cohorts, and 2-sample *t *tests, Wilcoxon signed-rank, and wilcoxon rank-sum test were performed for comparisons of continuous variables.

To determine predictors of initiation with duloxetine versus venlafaxine XR, a multivariate logistic regression model was used, with initiation of treatment with duloxetine versus venlafaxine XR as a binary dependent variable coded as 1 = duloxetine and 0 = venlafaxine XR. Covariates in the model included patient age (with age 18-35 years as a reference group), gender, prescriber specialty, dummy variables for prior medication use, and medical and diagnostic histories. Additional predictors included 3 derived indicators for prior uses of ≥3 unique antidepressants and ≥3 unique pain medications and patients with ≥8 unique disease classes. Adjusted odds ratios (ORs) and 95% confidence intervals (CIs) are presented to show the strengths of the associations with each significant predictor in the model. The reliability of the model was checked to evaluate predictability values, including receiver-operator characteristic (ROC) curves. All statistical analyses were performed using SAS version 9.1 (SAS Institute, Inc., Cary, NC). Tests were conducted at a two-tailed α = 0.05. Two-tailed p-values are presented unadjusted for multiplicity. However, as a total of 65 different covariates were examined, Hochberg's adjustment was computed and p-values of approximately 0.001 or less met the multiplicity adjusted level of statistical significance [21]. Thus, p-values of 0.001 or less are denoted as statistically significant in the tables.

## Results

A total of 18,155 patients with MDD met the selection criteria, including 9,641 (53%) patients initiating treatment with duloxetine and 8,514 (47%) patients initiating with venlafaxine XR. Patients in the two cohorts differed in sociodemographic characteristics (Table 1). Patients initiating treatment with duloxetine were older and slightly more likely to be female (*P *< 0.0001). Duloxetine was significantly more likely to be used in the eastern, southern, and western regions of the United States and venlafaxine XR in the Midwest. In addition, a higher proportion of patients initiating treatment with duloxetine (vs. venlafaxine XR) received prescriptions from psychiatrists (*P *< 0.0001).

**Table 1:** Demographic characteristics and health care provider for patients initiating therapy with duloxetine versus venlafaxine XR

Characteristic	Duloxetine Group (n = 9,641)	Venlafaxine XR Group (n = 8,514)	p-value
Age (y), mean ± SD	45.0 ± 11.4	42.4 ± 12.2	.0001
Age group (%)			.0001
18-35 y	20.9	29.3	
36-50 y	42.6	40.9	
51-65 y	36.5	29.8	
Female (%)	73.6	71.5	.001
Plan type (%)		(NS)	.061
Health maintenance organization	26.6	27.9	
Indemnity	5.4	4.7	
Preferred provider	49.9	48.7	
Point of service	16.1	16.3	
Other or unknown	2.0	2.4	
Region (%)			.0001
East	17.1	16.6	
Midwest	39.3	43.7	
South	24.1	21.4	
West	19.5	18.3	
Prescribed by psychiatrists (yes vs. no)	46.2	41.8	.0001

XR = extended-release. Indemnity - a type of fee-for-service medical plan that reimburses the patient and/or provider as expenses are incurred. All comparisons of demographics between cohorts are statistically significant (at least *P *< 0.001), except those marked by (NS = Not statistically significant).

### Prior Medication Use

Higher proportions of patients initiating treatment with duloxetine (vs. venlafaxine XR) previously received prescriptions for psychotropic medications, including SSRIs, TCAs, other antidepressants, anxiolytics, anticonvulsants, and antipsychotics(All significant P < .0001; Table 2). In addition, significantly higher proportions of patients initiating treatment with duloxetine previously received medications for painful conditions during the year before the index date. This finding was consistent (*P *< 0.0001 for each comparison) across each class of medications examined, including analgesics (63% vs. 51%), skeletal-muscle relaxants (27% vs. 17%), and antimigraine medications (12% vs. 9%). Other medication classes were also prescribed to higher proportions of patients initiating treatment with duloxetine compared with venlafaxine XR during the year before the index date, including hypnotics (30% vs. 22%) and antiulcer (29% vs. 23%) medications (*P *< 0.0001 for each comparison). Furthermore, a slightly higher proportion of patients in the duloxetine cohort in the previous year received ≥3 unique antidepressants (21% vs. 17%), and 26% of duloxetine patients received ≥3 unique pain medications compared with 16% for venlafaxine XR (*P *< 0.0001).

**Table 2:** Medication use in the prior 12-month period for patients initiating therapy with duloxetine versus venlafaxine XR.

Previous Medication Use	Duloxetine Group, % (n = 9,641)	Venlafaxine XR Group, % (n = 8,514)	p-value
Antidepressants			
SSRIs	59.5	52.7	.0001
TCAs	12.6	7.8	.0001
MAOIs	0.2	0.1 (NS)	.275
Other antidepressants	45.0	36.5	.0001
Anxiolytics	57.0	47.4	.0001
Benzodiazepines	53.8	44.5	.0001
Buspirone	4.8	4.1 (NS)	.015
Hydroxyzine	5.7	4.9 (NS)	.009
Anticonvulsants	30.1	17.9	.0001
Antipsychotics	20.0	15.4	.0001
Atypical	17.8	13.2	.0001
Typical	3.5	2.9 (NS)	.021
Analgesics	63.1	51.3	.0001
Skeletal-muscle relaxants	26.5	17.1	.0001
Antimigraine medications	12.3	9.2	.0001
Hypnotics	30.2	22.3	.0001
Other medications			
Antiulcer drugs	29.4	22.7	.0001
Antihyperlipidemics	20.0	15.5	.0001
Antihypertensives	17.9	13.9	.0001
Antiasthmatics	17.7	15.4	.0001
ADHD medications			
Stimulants	13.6	9.2	.0001
Atomoxetine	2.0	1.7 (NS)	.079
Antihistamines	10.3	6.7	.0001
β-Adrenoceptor blockers	8.9	7.3	.0001
Antidiabetics	9.0	6.7	.0001
Antimanics	3.0	2.3	.001
≥3 unique antidepressants	20.8	16.6	.0001
≥3 unique pain medications	25.5	15.6	.0001

XR = extended release; SSRIs = selective serotonin reuptake inhibitors; TCAs = tricyclic antidepressants; MAOIs = monoamine oxidase inhibitors; ADHD = attention-deficit/hyperactivity disorder. All comparisons of medication use between cohorts are statistically significant at least *P *< 0.001), except those marked by (NS = Not statistically significant).

### Comorbid Conditions

Slightly higher proportions of depressed patients treated with duloxetine compared with venlafaxine XR had prior diagnoses of comorbid medical conditions during the 12 months before the index date (Table 3). Patients initiating treatment with duloxetine were more likely to have ≥8 unique medical conditions compared with those treated with venlafaxine XR (39% vs. 29%; *P *< 0.0001). Individuals beginning treatment with duloxetine were also significantly more likely than those taking venlafaxine XR to have prior pain in the diagnostic subcategories evaluated, including muscle (56% vs. 43%), back (34% vs. 25%), head (25% vs. 21%), chest (20% vs. 17%), or other pain (27% vs. 23%) (*P *< 0.0001 for each comparison vs. venlafaxine XR). Those initiating treatment with duloxetine were also more likely to have prior fibromyalgia diagnosed than their counterparts initiating therapy with venlafaxine XR (17% vs. 10%; *P *< 0.0001).

**Table 3:** Comorbid medical conditions in the prior 12-month period for patients initiating therapy with duloxetine versus venlafaxine XR.

Condition	Duloxetine Group, % (n = 9,641)	Venlafaxine XR Group, % (n = 8,514)	p-value
Other depressive disorder	47.9	46.5 (NS)	.065
≥8 unique medical conditions	38.6	29.1	.0001
Pain	76.3	67.8	.0001
Muscle pain	56.1	43.1	.0001
Fibromyalgia	17.4	9.5	.0001
Back pain	34.2	24.8	.0001
Head pain	25.3	21.1	.0001
Chest pain	20.0	16.5	.0001
Neuropathic pain	6.6	3.2	.0001
Irritable bowel syndrome	5.2	3.9	.0001
Other pain	27.1	23.2	.0001
Dyslipidemia	30.1	25.3	.0001
Hypertension	28.9	23.2	.0001
Anxiety disorders	25.6	24.6 (NS)	.145
GAD	13.6	12.7 (NS)	.054
Panic anxiety	6.15	5.93 (NS)	.536
PTSD	5.02	4.36 (NS)	.035
Social anxiety	1.11	1.46 (NS)	.037
Other anxiety	6.05	5.95 (NS)	.794
Sleep disorder	21.2	16.8	.0001
Gastrointestinal	20.4	16.8	.0001
Nondependence drug abuse	12.4	12.7 (NS)	.439
Diabetes mellitus	11.5	8.6	.0001
Bipolar disorder	11.1	9.3	.0001
Asthma	10.1	8.9 (NS)	.006
Heart disease	6.0	4.7	.0001
Organic psychosis	5.9	4.4	.0001
ADHD	5.4	4.4 (NS)	.003
Drug dependence	5.5	4.4	.0006
Alcohol dependence	4.8	4.9 (NS)	.668
Diabetic neuropathy	2.0	1.3	.001
Schizophrenia	1.2	1.3 (NS)	.744

GAD = generalized anxiety disorder; PTSD, = post-traumatic stress disorder; ADHD = attention-deficit/hyperactivity disorder. All comparisons of comorbidities between cohorts are statistically significant (at least *P *< 0.001), except those marked by (NS = Not statistically significant).

### Predictors of Duloxetine Treatment

**Figure 1:**
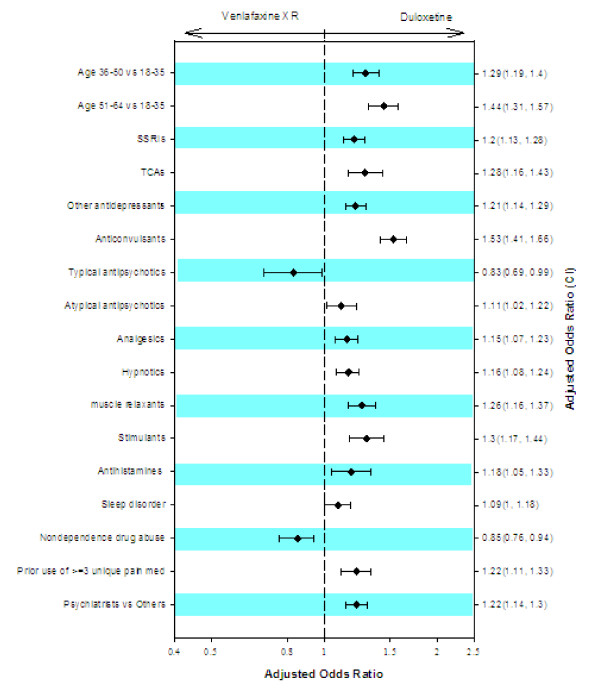
**Adjusted odds ratios and 95% confidence intervals for significant predictors of initiating treatment with duloxetine compared with venlafaxine XR**. XR = extended release; SSRIs = selective serotonin reuptake inhibitors; TCAs = tricyclic antidepressants; MAOIs = monoamine oxidase inhibitors; ADHD = attention-deficit/hyperactivity disorder.

Adjusted logistic regression modeling revealed pretreatment factors that uniquely predicted treatment with duloxetine or venlafaxine XR (Figure 1). Older patients (>35 years) were more likely to be initially treated with duloxetine compared with venlafaxine XR (*P *< 0.05). Patients were more likely to be treated with duloxetine if they had previously received prescriptions for SSRIs, TCAs, other antidepressants, anticonvulsants, atypical antipsychotics, analgesics, hypnotics, muscle relaxants, stimulants, or antihistamines, or if they had a diagnosis of sleep disorder during the previous 12 months (*P *< 0.05). Further, duloxetine treatment was significantly associated with previous prescriptions for ≥3 unique pain medications and with receiving prescriptions from a psychiatrist compared with other physicians. Initial treatment with venlafaxine XR was more likely in patients who had received prescriptions for typical antipsychotics (P = 0.037) or had a diagnosis of drug abuse (*P *= 0.001).

A sensitivity analysis for the logistic analysis was conducted to examine for the impact of continuous coding of the prior antidepressant, prior pain medication, and prior medical cormorbidity categories. Utilizing continuous versions of the variables did not change the inference (whether they are statistically significant or not) for these factors.

Model reliability checking supported the results of the regression analysis. Based on the logistic regression analysis using prior demographic characteristics, prescription by a psychiatrist, and diagnostic and prescription histories, it was possible to correctly predict 61% of patients who initiated treatment with duloxetine and 61% of patients who initiated treatment with venlafaxine XR. The area under the ROC curve (AUC) was 0.64.

### Health Care Costs

**Figure 2:**
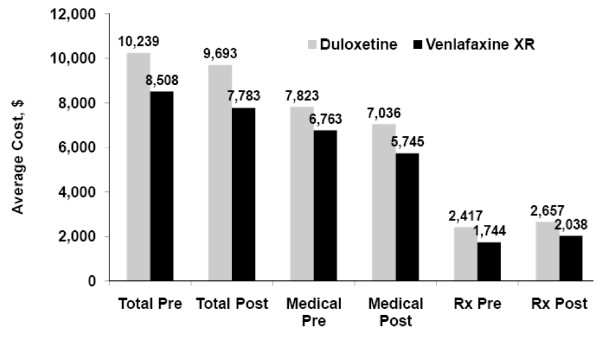
**Pre- and post- 6-month average cost for treatment with duloxetine versus venlafaxine XR (Wilcoxon signed-rank test for pre and post comparison)**. Total cost, medical cost and pharmacy cost are significantly changed from prior 6-month to post 6-month (P < 0.05). XR = extended release; Total = total cost; Medical = total medical cost; Rx = total pharmacy costs. All comparisons of cost between cohorts (Wilcoxon rank-sum test) are statistically significant (*P *< 0.005).

The distribution of health care costs in the 6 months before and 6 months after the index date is presented in Figure 2. On average, patients in the duloxetine cohort incurred significantly higher prior total health care costs (*P *< 0.005). The average prior 6-month total cost for patients initially treated with duloxetine was $10,239 and for patients treated with venlafaxine XR, $8,508. Although total pharmacy costs increased by more than $200 for each medication cohort in the subsequent 6 months (P < 0.001), average medical costs significantly decreased for each cohort (P < 0.001), resulting in a net average significant decrease in total health care cost in the 6 months after SNRI initiation of $546 for the duloxetine cohort and $725 for the venlafaxine XR cohort (P < 0.05).

## Discussion

This study examined existing clinical practice patterns of SNRIs duloxetine and venlafaxine XR to determine if, in usual clinical practice, they appear to be used as interchangeable medications or if there is evidence of targeted use of each for more specific MDD patient populations. The evidence favors targeted use: logistic regression prediction of treatment choice from pretreatment characteristics is substantially higher than 50%, which would be expected for a random choice between the 2 SNRIs. In our logistic regression analysis, 61% of patients receiving initial prescriptions for duloxetine and 61% for venlafaxine XR were predictable from pretreatment characteristics.

Patients with MDD who were initially treated with duloxetine tended to be older and to have more complex clinical presentations compared with their counterparts treated with venlafaxine XR. During the year before initiating SNRI treatment, future duloxetine patients were more likely than future venlafaxine XR patients to have a history of comorbid psychiatric and nonpsychiatric conditions, including pain. More duloxetine initiators were also likely to have previously taken other medications for mood, pain, and other medical conditions and were more likely to have received prescriptions for ≥3 distinct antidepressants in the previous year. As would be expected with a greater comorbid disease burden, patients commencing therapy with duloxetine were also more likely to have had higher previous health care resource utilization and total health care costs compared with the venlafaxine XR cohort. Use of either medication was associated with a net reduction in total medical costs in the 6 months after their initiation, despite higher pharmacy costs.

These findings of substantial differences in pretreatment case mixes and costs between patients initiating treatment with either of the two SNRIs in actual practice patterns appear to be at odds with what might be expected based on their efficacy with each other and with the SSRIs, as reported in clinical trials and comparative effectiveness meta-analyses [13,14]. We suspect that a reason for this apparent discrepancy of trial data from real-world use is the emerging clinical practice pattern of successive step therapy [22,23], as most prominently illustrated in the Sequenced Treatment Alternatives to Relieve Depression (STAR-D) effectiveness trial [9]. The STAR-D studies mimic real-world care by initiating treatment with SSRI in all patients, then offering nonremitting patients recommended second-step and third-step treatments. In STAR-D [2], nonremitting patients requiring additional treatment steps were predicted by many of the same pretreatment characteristics reported herein: prior treatment nonresponse, multiple comorbid medical conditions, multiple psychiatric comorbidities, as well as more severe depressive symptoms (data not available in this study). In STAR-D terms, patients taking SNRIs and examined in this study appear to resemble nonremitting, complex, Step 2 and Step 3 patients. Thus, in contrast to clinical trials, which randomize average patients to different treatments, SNRIs are more often initially prescribed to patients with a more complex and challenging clinical picture in usual clinical care. Use of the SNRIs for complex patients is in line with emerging studies suggesting that SNRIs may be particularly effective in patients with a more severe and complicated initial clinical presentation who are most prone to fail first-line SSRI treatment [6-8].

To best of the authors' knowledge, this is the first study to assess the association of demographics, prior comorbidities, medication use, and treatment cost, with treatment initiation for duloxetine and venlafaxine XR in the usual care of patients with MDD. The findings demonstrated that duloxetine is primarily used for patients with a complicated disease and medication profile.

### Potential Study Limitations

As with most other retrospective administrative claims data analyses, this study is subject to potential limitations, including selection biases and unmeasured confounding factors. We examined individuals who were commercially insured and had 12 months of continuous enrollment; patients who did not meet these inclusion criteria were excluded. We also selected the most recent initiation of SNRIs in patients with MDD diagnoses, and this method diverges from the usual practice of starting with an incident diagnosis and having the first medication define an index case in claims studies. Data on MDD severity, treatment outcomes, patient preferences, and health plan restrictions were also unavailable; hence, comorbidities and health care resource utilization serve as indirect indicators of disease severity and outcome. Although our logistic regression models adjusted for certain confounding factors, potential biases may still exist because of these and other unmeasured factors. The potential confounding impacts of local step-therapy guidelines, and personal insurance drug tiers could also not be examined with available data. The timeframe for the data in this analysis started one year after duloxetine was on the market in order to eliminate 'early adopters' of a new medication and better capture a representative pattern in usual care settings. However, it is unclear if some of the differences observed here were due to the fact that duloxetine was still the newer antidepressant at this time and whether the observed factors would persist if later data was utilized. Greater clinical severity and low initial dosing have been found to be factors predicting earlier antidepressant switching [24] and such patients may have preferentially tried the newer medication. We did not assess initial dose in this work and additional confirmatory work utilizing more recent data is warranted. Finally, the descriptive cost changes over time may reflect regression to the mean that could be common to any added treatment rather than cost-offsets unique to SNRIs. In the light of these potential limitations, the results of this study need to be further replicated and extended using different types of studies in broader patient and medication populations and with direct clinical data to aid in examining the impacts of depression severity and outcomes [19,25].

### Clinical Implications

Initial SNRI care for patients with more complex MDD resembles a targeted approach in which clinicians draw subtle distinctions between duloxetine and venlafaxine XR. Duloxetine appears to be prescribed for somewhat more complicated and costly patients than venlafaxine XR. These treatment patterns suggest that both medications have a place on formularies to allow for optimal patient matching, especially for patients not responding to initial step therapy choices. Effectiveness studies such as STAR-D [2] and Randomized Trial Investigating SSRI Treatment (ARTIST) [26] demonstrate that most patients' MDD does not remit with initial SSRI treatment, and that the need for second-stage or higher steps of care represent the norm rather than the exception. While clinical guidelines [11] tend to focus on first-line recommendations, less is known about optimal choices and targeting of patients needing treatment changes after initial nonremission. There is also little evidence-based guidance supporting optimal initial matching to minimize nonremission and the risk that patients will either discontinue treatment or refuse an alternative regimen when first-line care fails.

## Conclusions

Patients with MDD receiving initial treatment with duloxetine were more likely to be older and to have comorbid medical conditions and complex prior medication treatment histories compared with their counterparts initially receiving venlafaxine XR. Duloxetine initiators were also more likely to have been under prior psychiatric care, to have ≥8 unique medical conditions, and to have used ≥3 unique pain medications within the year before initiating SNRI treatment. Further research in more heterogeneous patient populations may delineate more definitively the potential patient-related outcomes and treatment cost consequences associated with more optimally targeted SNRI treatments.

## Competing interests

This study and its report were supported and fully funded by Eli Lilly and Company (Indianapolis, Indiana), which had a role in study design; data acquisition and analysis; preparation and revision of the manuscript; and the decision to publish the findings. Wenyu Ye, Yang Zhao, Rebecca L. Robinson, Ralph W. Swindle are full-time employees and minor shareholders of Eli Lilly and Company, Indianapolis, IN.

## Authors' contributions

Concept and design WY, YZ, RR, RWS; acquisition of data WY, YZ, RR; analysis and interpretation of data WY, YZ, RR, RWS; drafting of the manuscript WY, RWS; critical revision of the manuscript for important intellectual content WY, YZ, RR, RWS; statistical analysis WY; obtaining funding WY; and supervision RWS. All authors have read and approved the final manuscript.

## Appendix

List of 17 medical categories; Table 4 (used for patients' unique medical conditions)

**Table 4:** Medical Condition ICD-9-CM Code

Infectious and parasitic diseases	001-139
Neoplasms	140-239
Endocrine	240-279
Blood	280-289
Mental	290-319
Nervous system and sense organs	320-389
Circulatory	390-459
Respiratory	460-519
Digestive	520-579
Genitourinary	580-629
Childbirth	630-677
Skin	680-709
Musculoskeletal system	710-739
Congenital	740-759
Perinatal	760-779
Other illness	780-799
Injury poison	800-999

ICD-9-CM = *International Classification of Diseases*, 9th Edition.

## Pre-publication history

The pre-publication history for this paper can be accessed here:

http://www.biomedcentral.com/1471-244X/11/19/prepub
